# Application of juniper (*Juniperus communis*) essential oil nanoemulsions to control spoilage and pathogenic bacteria in fish

**DOI:** 10.3389/fmicb.2026.1758540

**Published:** 2026-01-22

**Authors:** Hakan Çolak, Mustafa Durmuş, Esmeray Küley, Ali Rıza Köşker, Yetkin Sakarya, Tuba Esatbeyoglu, Fatih Özogul

**Affiliations:** 1 Department of Seafood Processing Technology, Faculty of Fisheries, Cukurova University, Adana, Türkiye; 2 Department of Molecular Food Chemistry and Food Development, Institute of Food and One Health, Gottfried Wilhelm Leibniz University Hannover, Am Kleinen Felde, Hannover, Germany; 3 Biotechnology Research and Application Center, Cukurova University, Adana, Türkiye

**Keywords:** antimicrobial activity, fish spoilage bacteria, foodborne pathogens, juniper essential oil, nanoemulsion

## Abstract

This study evaluated the antimicrobial activity of pure juniper essential oil and its nanoemulsion formulations (2, 4, and 6%) against five foodborne and fish spoilage bacterial species, including *Staphylococcus aureus, Salmonella Paratyphi* A, *Vibrio vulnificus*, *Photobacterium damselae* and *Proteus mirabilis*. The GC–MS profile of pure juniper essential oil (EO) revealed thirty components, including *α*-pinene, which accounted for 90.05% of the total volatiles. The antimicrobial activity was studied by measuring the inhibition zone diameters by the agar well diffusion method and determining the minimum inhibitory concentration (MIC) and minimum bactericidal concentration (MBC) values by micro dilution method. A clear dose–response relationship was observed in nanoemulsion formulations; as EO concentration increased, inhibition zones enhanced and MIC/MBC values decreased. *S. aureus* showed inhibition even at 2%JNEO (~15 mm), reaching a maximum of 22.1 mm at 6%JNEO. Among Gram-negative pathogens, *Vibrio vulnificus* showed the highest susceptibility, particularly to pure juniper essential oil, as reflected by low MIC and MBC values. *P. damselae* and *S. paratyphi* A exhibited intermediate susceptibility (MIC 1.56–12.5 mg/mL; MBC 12.5–25 mg/mL), while *P. mirabilis* showed high resistance (MIC 12.5 mg/mL; MBC > 100 mg/mL) and only limited inhibition. Among the tested bacteria, *Staphylococcus aureus* and *Vibrio vulnificus* showed the highest susceptibility, with inhibition zones and MIC/MBC values decreasing in a concentration-dependent manner. This antimicrobial activity may be associated with the high *α*-pinene content of juniper essential oil. These results highlight the potential of juniper essential oil nanoemulsions as effective natural preservatives to control fish spoilage and foodborne bacteria in the seafood industry.

## 1 Introduction

The food industry continues to grow in line with the rapidly increasing global population and changing consumer expectations. Today, consumers want to access food products that are not only tasty and nutritious, but also safe, pathogen-free and have a longer shelf life (Mei et al., 2019; Faheem et al., 2022). However, problems caused by pathogenic bacteria, fungi and unpleasant odours or flavours during the production and storage of food products constitute a significant barrier to food safety and quality protection. These problems are particularly important for rapidly perishable food products such as fish. Fish spoilage bacteria are one of the main factors that shorten the shelf life of fresh fish and controlling these bacteria is critical both to protect consumer health and to reduce economic losses. For this purpose, the use of preservatives with antimicrobial properties has become widespread. Although the use of synthetic preservatives is higher than natural preservatives in industrial scale, in recent years, there has been a trend towards the use of natural alternatives instead of synthetic preservatives in the food industry in line with consumer expectations (Mei et al., 2019; Alami El Hassani et al., 2025). One of the main reasons for this trend is the negative effects of synthetic chemicals on human health and the environmental problems they create. For these reasons, natural preservatives are used in many new technologies for natural prevention against food spoilage (Alami El Hassani et al., 2025). In particular, natural protection strategies based on biologically active compounds of plant origin come to the fore. For this purpose, herbal products with significant antimicrobial and antioxidant activity are widely preferred as natural preservatives. Among these natural preservatives, plant essential oils (EO) stand out due to their strong antimicrobial and antioxidant properties. EO of plants contain a series of terpenes that provide these activities (Faheem et al., 2022; Teshome et al., 2022). EOs are complex, volatile mixtures of compounds derived from parts of the plant such as leaves, flowers, seeds, roots and bark, containing components such as terpenes (e.g., thymol, carvacrol), phenols (e.g., eugenol) and alcohols (e.g., linalool).

One of the promising EOs in terms of food preservation is juniper EO, which is mainly obtained from *Juniperus communis* species. Juniper EO is a natural product with a high content of monoterpenes and sesquiterpenes. Compounds such as *α*-Pinene, Sabinene and *β*-Pinene are prominent in the chemical content of the oil. These compounds make up a large part of the total content of the oil and are known for their antimicrobial, antioxidant and anti-inflammatory effects (Aloui et al., 2021). The chemical content may vary depending on the species of juniper plant, its geographical origin, growing conditions and the extraction method applied. However, these chemical components of juniper EO increase its potential as a natural antimicrobial agent for food safety and preservation studies (Ložienė and Labokas, 2012). In particular, the *α*-pinene content shows strong antimicrobial effects against both Gram-positive and Gram-negative bacteria, while components such as sabinene can increase bacterial membrane permeability.

Despite this broad biological effect of EOs, their direct application to food systems presents some challenges. Their low water solubility, high volatility and pungent flavours may affect sensory properties and reduce consumer acceptance. Furthermore, inhomogeneous dispersion and rapid evaporation of EOs may also limit their preservative efficacy (da Silva Martins et al., 2023). In order to overcome these limitations, nanoemulsion technology has been developed to increase the solubility, stability and bioavailability of EOs (McClements et al., 2009).

Nanoemulsions are kinetically stable colloidal systems consisting of droplets usually below 200 nm, containing oil and water phases, stabilised by surfactants. This structure allows EO to interact more effectively with microbial membranes (Donsì and Ferrari, 2016). Nanoemulsion technology stands out as a highly effective method to increase the biological activity of oils. Thanks to their small droplet size, nanoemulsions offer a large surface area, which increases the effect of EO on target microorganisms. Preparation of nanoemulsions containing juniper EO at different ratios and evaluation of their antimicrobial effects on fish spoilage bacteria are especially important for extending the shelf life and maintaining the quality of fresh fish (Donsì and Ferrari, 2016). Although essential oil nanoemulsions based on thyme, citrus, and other plant sources have been widely investigated, information on the antimicrobial potential of juniper (*Juniperus communis*) essential oil nanoemulsions against fish spoilage bacteria and seafood-related pathogens remains limited. In particular, the combined evaluation of chemical composition, nanoemulsion physicochemical properties, and *in vitro* antimicrobial efficacy against marine-origin bacteria represents a distinct aspect of the present study. In this context, this study aimed to evaluate the antimicrobial activity of pure juniper EO and its nanoemulsion formulations at different concentrations against key fish spoilage bacteria and foodborne pathogens. The findings will contribute to the development of natural preservation strategies for the seafood industry.

## 2 Materials and methods

### 2.1 Material

Juniper (*Juniperus communis*) essential oil was obtained from a commercial supplier located in Antalya, Türkiye. The oil was produced by steam distillation of juniper berries and stored in amber-colored glass bottles at 4 °C until further analysis.

### 2.2 Preparation of nanoemulsions

Nanoemulsions were prepared by modifying the method proposed by Joe et al. (2012). Different proportions of juniper EO (2, 4 and 6%), distilled water and 4% Tween 80 (Sigma Aldrich, Taufkirchen, Germany) were used for nanoemulsion formulation. In all nanoemulsion formulations, the concentration of Tween 80 was kept constant at 4%, while the essential oil concentration (2, 4, and 6%) was adjusted by proportionally modifying the aqueous phase.

The components were mixed under appropriate conditions to obtain a homogeneous pre-emulsion. The aqueous phase containing the surfactant was incubated at 86 °C for 1 h to facilitate homogenization and promote nanoemulsion formation, rather than to thermally treat the essential oil. The formulation was maintained under controlled, closed conditions to minimize potential volatilization losses. Gas chromatography–mass spectrometry (GC–MS) analysis was performed on the pure juniper essential oil prior to the nanoemulsion preparation process. After the incubation period, the mixture was cooled to room temperature and pre-sterilized distilled water was added to the cooled mixture at the determined ratios. Homogenization of the nanoemulsions was performed using an ultrasonic probe homogenizer (CY-500 sonicator, Optic Ivymen System, Barcelona, Spain). The homogenization process was applied for 15 min at 95% amplitude. Ultrasonication was performed in continuous mode without pulse application, and the samples were kept in an ice bath during processing while the temperature was continuously monitored to prevent excessive heating. The ultrasonic device used operates at a power of 500 W and a frequency of 20 kHz and includes a ¼ inch titanium alloy probe with a piezoelectric transducer (diameter: 5.6 mm; height: 60 mm). This process was applied in order to reduce the droplet size of the nanoemulsions and ensure a stable structure.

### 2.3 Physical properties of nanoemulsions

Physicochemical properties of the nanoemulsions were analysed at the METU Central Laboratory (Middle East Technical University, Ankara, Turkey). The droplet size of the emulsions was determined using a Mastersizer 2000 (Malvern Instruments, Malvern, UK) based on laser diffraction principles. The polydispersity index (PDI) was measured by dynamic light scattering (DLS) using a Zetasizer Nano-ZS (Malvern Instruments, Malvern, UK), following the methodology described by Hosseinnia et al. (2017).

### 2.4 Food-borne pathogens and fish spoilage Bacteria

In the study, Gram positive *Staphylococcus aureus* (ATCC29213) and Gram negative *Salmonella Paratyphi* A (NCTC13) were used as food-borne pathogenic bacteria. *Photobacterium damselae, Proteus mirabilis* and *Vibrio vulnificus* were also used as spoilage bacteria. These bacteria were isolated from spoiled anchovy, mackerel and sardine meat (Kuley et al., 2017). These bacteria were selected to test the antimicrobial activity of pure juniper EO and its nanoemulsions, and the study objectives were achieved by analysing fish spoilage and foodborne pathogenic microorganisms.

### 2.5 Antimicrobial activity analysis

Antimicrobial activity analysis was carried out to evaluate the effects of juniper EO and nanoemulsions prepared using this oil on foodborne pathogenic and fish spoilage bacteria. These analyses were performed using different methods and aimed to verify and comparatively evaluate the antibacterial activity of nanoemulsions. Within the scope of the analysis, standard methods such as Agar Well Diffusion Assay, MIC and MBC were applied.

### 2.6 Agar well diffusion method

In this study, the antimicrobial activity of juniper EO and its nanoemulsions was determined using Mueller-Hinton Agar (MHA, Merck 1.05437, Darmstadt, Germany) according to the well diffusion method of Hwanhlem et al. (2017). Test bacteria were grown in Nutrient broth (Merck 1.05443, Darmstadt, Germany) at 37 °C for 24 h and standardised to 0.5 McFarland cell density (108). Each bacterial cell culture (100 μL) was inoculated into a petri dish containing 20 mL of Muller-Hinton agar. Five 5 mm wells were made in solid medium. Four wells were inoculated with 50 μL of juniper EO in pure or nanoemulsion form. Distilled water was transferred to the other well as control. The negative control was Tween 80. Petri dishes were then incubated at 37 °C for 24 h. After incubation, the zones of inhibition formed around each well were measured in mm using callipers.

### 2.7 Minimum inhibition concentration (MIC) and minimum bactericidal concentration (MBC)

MIC and MBC values of pure juniper EO and its nanoemulsions were determined using the CLSI (Clinical and Laboratory Standards Institute, 2006) microdilution method. MIC is defined as the lowest concentration at which the tested substance completely stops the growth of the microorganism, while MBC is the lowest concentration that completely kills the microorganism. The bacterial cultures tested for the experiments were prepared according to the 0.5 McFarland standard and studied using Mueller-Hinton Broth (MHB) medium. For antimicrobial tests, 50 mg/mL stock solutions of juniper EO and nanoemulsions were prepared and diluted to 0.19 μg/mL by the serial dilution method. To each dilution, 1 mL of bacterial suspension was added, and the prepared tubes were incubated at 37 °C for 24 h. After incubation, the turbidity in the tubes was observed, and the lowest concentration without growth was recorded as the MIC value. Following the determination of the MIC values, liquid cultures from the tubes with no growth were inoculated onto Mueller-Hinton agar and incubated at 37 °C for another 24 h. As a result of this second incubation, the lowest concentration at which no bacterial growth was observed was recorded as the MBC value. The reported MIC and MBC values represent the final concentrations of juniper essential oil and nanoemulsion formulations in the test system after dilution and inoculation.

### 2.8 Statistical analyses

Statistical analyses were performed using one-way analysis of variance (ANOVA) followed by Duncan’s Multiple Comparison Test (SPSS 22, Chicago, Illinois, USA) for *post hoc* mean separation among treatment groups. All experiments were conducted in triplicate as independent experimental repeats, and data are presented as mean ± standard deviation. Differences were considered statistically significant at *p* < 0.05.

## 3 Results and discussion

### 3.1 Physicochemical analysis of nanoemulsions

The average droplet size, polydispersity index (PDI) and surface tension values of the nanoemulsions are presented in Table 1. In the JNEO group with 2% EO content, the relatively low oil phase content resulted in more effective surfactant coverage during the droplet formation process and thus the smallest average droplet size of 21.89 nm was reached. When the essential oil content was increased to 4%JNEO, droplet deposition was promoted due to the increase in viscosity and change in the surfactant/oil phase ratio, resulting in the largest average droplet size of 377.9 nm. PDI values ranged from 0.006 to 0.420; higher PDI values indicated a decrease in the homogeneity of the droplet distribution. The higher PDI value indicates a broader droplet size distribution, suggesting increased polydispersity related to formulation composition. The surface tension measurements decreased to 41.38 mN/m in the JNEO group containing 6% EO, which is attributed to the fact that with the increase in the amount of oil phase, the surfactants form a more organized layer at the water/oil interface, thus reducing the surface tension. The highest surface tension value was 44.24 mN/m in the 2%JNEO group due to the interface instability caused by the low oil phase content. The results obtained in the present study both parallel and differ from the nanoemulsion properties reported in the literature. Joe et al. (2012) reported that the droplet sizes of nanoemulsions prepared from commercial oils such as sunflower seed, castor oil, coconut, peanut and sesame oil ranged between 72.52–875.22 nm; this wide range is thought to be due to the differences in formulation parameters, surfactant types and ratios used in the study. Similarly, some studies have reported nanoemulsions and nanoemulsion-based systems with droplet sizes extending up to approximately 1,000 nm, depending on the formulation composition, oil concentration, and preparation conditions (Costa et al., 2020; Gul et al., 2022; Wilson et al., 2022). Accordingly, the formulations obtained in this study can be defined as nanoemulsion-based systems exhibiting nano-scale properties. Yazgan (2013) reported that sunflower seed oil-based nanoemulsion reached an average droplet size of 381.99 nm; the similarity here is due to the fact that the amount of oil phase directly affects the droplet size. Özogul et al. (2017) reported that nanoemulsions obtained from thyme, rosemary, laurel and sage essential oils formed droplets of 112.82, 63.02, 66.02 and 59.48 nm in size, respectively; Durmus (2020) reported 47.40–94.66 nm droplet size and 35.15–39.18 mN/m surface tension values in formulations based on citrus essential oils (orange, mandarin, grapefruit, lemon). These differences are due to variations in the types of oils used, surfactant selection and nanoemulsion preparation methods.

**Table 1:** Properties of nanoemulsions based on essential oils.

	2%JNEO	4%JNEO	6%JNEO
Droplet size (nm)	21.89	377.9	330.1
Polydispersity index	0.420	0.066	0.006
Surface tension (mN/m)	44.24 ± 0.18	43.17 ± 0.24	41.38 ± 0.22

2%JNEO means juniper nanoemulsion at 2% concentration; 4%JNEO means Juniper nanoemulsion at 4% concentration; 6%JNEO means Juniper nanoemulsion at 6% concentration.

### 3.2 GC–MS results of juniper essential oil (EO)

The GC–MS results presented in Table 2 formed the basis for explaining the chemical composition of juniper essential oil and the potential antimicrobial effects of these components. As a result of the analyses, a total of 30 different compounds were identified, of which the main component was *α*-Pinene with 90.05%. The high α-Pinene content may have increased the effectiveness of the oil by damaging the microbial cell membrane, as this compound is known to show strong antibacterial and antifungal activity in the literature (Falcão et al., 2018; Najar et al., 2020). Indeed, the presence of other monoterpenes such as *α*-Myrcene (1.80%) and 2-*α*-Pinene (1.37%) may have synergistically interacted with this main component to broaden the antimicrobial spectrum.

**Table 2:** Components of juniper EO and GC–MS results.

Number	Compounds detected	% Amount	Measurement limit
1	Tricyclene	0.14	0.01%
2	α-Pinene	90.05	0.01%
3	2-α-Pinene	1.37	0.01%
4	α-Myrcene	1.80	0.01%
5	Delta-3-carene	0.10	0.01%
6	p-Cymene	0.27	0.01%
7	dl-Limonene	1.18	0.01%
8	ç-Terpinene	0.59	0.01%
9	α-Terpinolene	0.69	0.01%
10	α-Pinene oxide	0.01	0.01%
11	Thujone	0.01	0.01%
12	α-Campholene Aldehyde	0.14	0.01%
13	Trans-Pinocarveol	0.20	0.01%
14	Camphor	0.07	0.01%
15	Verbenol	0.03	0.01%
16	Myrtenal	0.03	0.01%
17	Berbenone	0.07	0.01%
18	Carveol	0.03	0.01%
19	Bornyl acetate	0.18	0.01%
20	Tetradecane	0.05	0.01%
21	α-Copaene	0.05	0.01%
22	ç-Muurolene	0.05	0.01%
23	trans-Caryophyllene	0.07	0.01%
24	Widdrene	0.05	0.01%
25	Germacrene-D	0.22	0.01%
26	α-Humulene	0.03	0.01%
27	α-Cadinene	0.11	0.01%
28	Veridiflorol	0.05	0.01%
29	α-Cedrol	1.23	0.01%
30	Other compounds	1.15	0.01%
	TOTAL	100	

The α-Cedrol (1.23%) and dl-Limonene (1.18%) contribute to keep juniper oil active against both bacterial and fungal pathogens. In particular, the known antimicrobial effect of *α*-Cedrol has been enhanced by the presence of this compound. On the other hand, compounds such as Thujone, α-Pinene oxide and Verbenol, although present in the range of 0.01–0.03%, can contribute to the overall antimicrobial activity by altering microorganism cell wall permeability even at low concentrations.

Although the obtained α-Pinene content of 90.05% is in line with the *α*-Pinene content of 77.11% previously reported in *Juniperus sabina* L. *cones* (Ertaş et al., 2019), it showed a higher value depending on the study species and regional ecological conditions. For example, in *Juniperus oxycedrus* L. subsp. oxycedrus, α-Pinene was found to be 32.85% and *β*-Myrcene 57.14%, while α-Pinene 19.7% and epi-Sedrol 17.8% were reported in oil obtained from *Juniperus excelsa* leaves (Gülsoy and Çıvğa, 2016; Doğan et al., 2016). These differences indicate that genetic diversity and geographical conditions among juniper species directly affect essential oil composition.

In conclusion, the component distribution in Table 2 shows that the high content of α-Pinene in juniper essential oil as well as the low concentrations of various monoterpenes and sesquiterpenes support the antimicrobial activity of the oil both directly and through synergistic mechanisms. This facilitated the interaction of the oil with microbial membranes in nanoemulsion formulations, leading to a strong inhibitory effect even at low concentrations.

### 3.3 Inhibition zone diameters of samples

Inhibition zone diameters measured using the agar well diffusion method (Table 3) clearly demonstrate the antimicrobial activity of juniper essential oil (EO) and its nanoemulsion formulations (JNEO) against the tested bacterial strains. In the group where EO was directly applied, the highest inhibition zone diameters were recorded, indicating more effective disruption of bacterial membrane integrity (*p* < 0.05). Since Tween 80 lacks antimicrobial qualities, it did not exhibit any inhibition zone.

**Table 3:** Inhibition zone diameters (mm) of juniper EO and nanoemulsion formulations on bacterial species.

Bacterium	Tween 80	Juniper EO	Nanoemulsions		
			2%JNEO	4%JNEO	6%JNEO
*Staphylococcus aureus*	ND	Full inhibition	8.75 ± 0.65^b^	9.00 ± 0.71^b^	17.00 ± 0.82^b^
*Salmonella Paratyphi* A	ND	Full inhibition	6.00 ± 0.00^b^	6.25 ± 0.50^b^	9.25 ± 0.50^a^
*Vibrio vulnificus*	ND	Full inhibition	52.25 ± 1.26^b^	54.25 ± 2.22^b^	64.00 ± 3.46^a^
*Photobacterium damselae*	ND	Full inhibition	15.50 ± 0.58^c^	25.00 ± 1.41^b^	35.75 ± 1.89^a^
*Proteus mirabilis*	ND	52.00 ± 2.45^a^	6.00 ± 0.00^c^	7.25 ± 0.50^bc^	8.33 ± 0.47^b^

* Mean value±Standard deviation (*n* = 3). Full inhibition: no visible bacterial growth on the agar surface. There is a significant difference (*p* < 0.05) between the groups in terms of values indicated with different letters (a–c) in the same row. 2%JNEO means Juniper nanoemulsion at 2% concentration; 4%JNEO means Juniper nanoemulsion at 4% concentration; 6%JNEO means Juniper nanoemulsion at 6% concentration. ND. Not detected.

**Figure 1:**
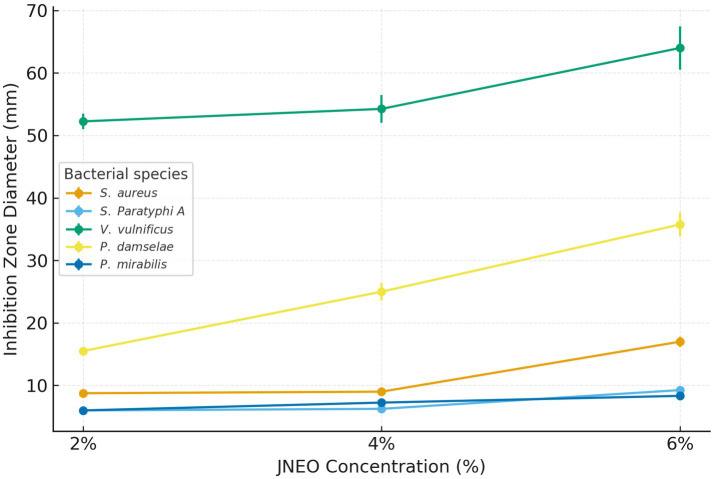
Dose–response curves showing the inhibition zone diameters (mean ± SD) of juniper essential oil nanoemulsions (JNEO) at different concentrations (2, 4, 6%) against selected fish spoilage bacteria. The antibacterial activity increased in a concentration-dependent manner, and the most pronounced effect was observed for *Vibrio vulnificus* and *Photobacterium damselae*.

As shown in Figure 1, inhibition zones increased in a concentration-dependent manner across all tested bacteria, particularly in *Vibrio vulnificus* and *Photobacterium damselae*. For *V. vulnificus*, the inhibition zone diameter increased from 52.25 ± 1.26 mm at 2% JNEO to 64.00 ± 3.46 mm at 6% JNEO, while for *P. damselae*, zone diameters increased from 15.50 ± 0.58 mm to 35.75 ± 1.89 mm, respectively.

In *Staphylococcus aureus* and *Salmonella Paratyphi A*, weak inhibition effects were observed at 2 and 4% JNEO concentrations, whereas at 6%, zone diameters reached 17.00 ± 0.82 mm and 9.25 ± 0.50 mm, respectively. These increases are attributed to the higher concentration of active monoterpenes in EO, which enhances their interaction with bacterial cell wall components. *Proteus mirabilis* showed high susceptibility to the pure essential oil, while its response to the nanoemulsion formulations was comparatively lower (Table 3).

Similar findings were reported by Taşar et al. (2022), who observed inhibition zones ranging from 8.13 ± 0.67 to 13.43 ± 0.31 mm for *Staphylococcus aureus* using *Juniperus excelsa* and *J. foetidissima* leaf oils, and by Aslan et al. (2021), who reported inhibition zones up to ≥50 mm depending on oil composition and bacterial strain.

The inhibitory effect varied depending on bacterial species and EO concentration. At higher concentrations, JNEO exhibited pronounced antibacterial effects against *V. vulnificus* and *P. damselae*, while *P. mirabilis* showed high susceptibility to the pure essential oil but comparatively lower responses to the nanoemulsion formulations.

The antibacterial efficacy of nanoemulsion formulations was frequently less potent than that of pure EO, despite improvements in transport and stability. Furthermore, in line with previous research, the inhibitory effect of nanoemulsions on bacteria grew as the EO concentration rose (Kayiran et al., 2025). In this work, 2, 4%, or 6% of the EO were incorporated into EO-based nanoemulsions. As a result, their levels of bioactive EO molecules are decreased. This could account for the higher antibacterial activity of pure EO compared to the nanoemulsion form (Özogul et al., 2021). Increased inhibitory zone is seen when the data are recalculated to account for the actual amount of pure EO (Zahi et al., 2017). Therefore, considering the EO content of the nanoemulsions, the antimicrobial efficiency of the nanoemulsion system increased with the addition of EO. These findings suggest that juniper EO and their nanoemusions forms can be considered a promising natural antimicrobial agent, particularly against Gram-negative marine bacteria, and that concentration control plays a critical role in optimizing formulation efficacy.

### 3.4 Minimum inhibition concentration (MIC) and minimum bactericidal concentration (MBC) results

**Figure 2:**
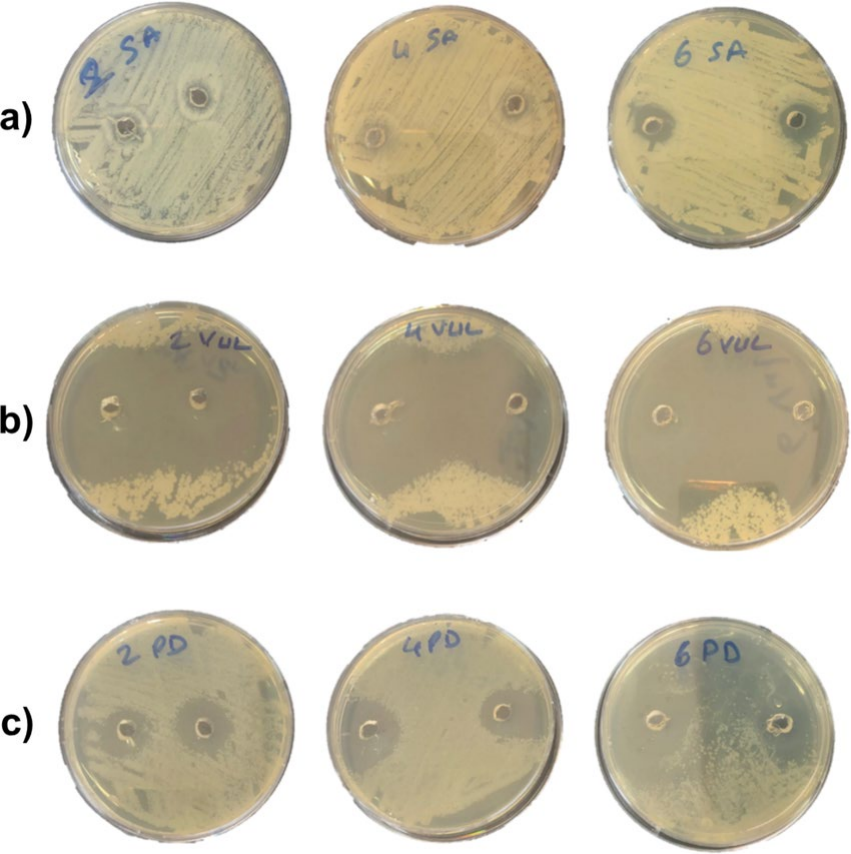
Antibacterial effect of juniper essential oil nanoemulsions (JNEO) at different concentrations (2, 4, and 6%) on foodborne and spoilage bacteria using agar well diffusion assay. **(a)** *Staphylococcus aureus*—JNEO caused a concentration-dependent increase in inhibition zone diameters. **(b)** *Vibrio vulnificus*—Wide and well-defined zones were observed, especially at higher concentrations. **(c)** *Photobacterium damselae*—Lower sensitivity was recorded, with smaller inhibition zones.

MIC and MBC values of Juniper EO and nanoemulsions on five different bacterial strains tested are given in Table 4. Tween 80 showed no bactericidal or inhibitory effects on any examined bacteria. MIC and MBC values of Juniper EO differ according to bacterial species. Against *Staphylococcus aureus*, the MIC and MBC of juniper EO was 0.78 6.25 mg/ mL, whilst towards *S. paratyphi* A, it exerted the respective MIC and MBC of 12.5 and 25 mg/ mL Juniper EO exhibited similar MIC and MBC against *Vibrio vulnificus* and *Photobacterium damselae*, with respective value of 1.56 and 12.5 mg/mL, indicating that the effectiveness of juniper EO on these species was high (Figure 2).

**Table 4:** MIC and MBC values (mg/mL) of Juniper EO and its different concentrations on bacterial species.

Bacteria type	Tween 80		Juniper EO		Nanoemulsions					
					2%JNEO		4%JNEO		6%JNEO	
	MIC	MBC	MIC	MBC	MIC	MBC	MIC	MBC	MIC	MBC
*S. aureus*	>100	>100	0.78	6.25	50	100	50	100	25	50
*S. paratyphi A*	>100	>100	12.5	25.0	100	>100	100	>100	50	>100
*Vibrio vulnificus*	>100	>100	1.56	12.5	50	100	50	100	12.5	50
*Photobacterium damselae*	>100	>100	1.56	12.5	100	>100	50	>100	50	100
*Proteus mirabilis*	>100	>100	12.5	12.5	100	>100	100	>100	50	>100

*2%JNEO means Juniper nanoemulsion at 2% concentration; 4%JNEO means Juniper nanoemulsion at 4% concentration; 6%JNEO means Juniper nanoemulsion at 6% concentration.

In, the MIC value was 1.56 mg/mL and the MBC value was 12.5 mg/mL and a completely bactericidal effect was observed as the concentration increased. In *Proteus mirabilis*, the MIC value was 12.5 mg/mL and MBC value was >100 mg/mL and it is understood that this species shows comparatively lower susceptibility to the nanoemulsion formulations, despite its clear sensitivity to the pure juniper EO. As a result of MIC and MBC analyzes, the following findings stand out:

*S. aureus* and *V. vulnificus* species are the most susceptible bacteria to juniper EO. Effective inhibition was achieved with low MIC values (0.78–1.56 mg/mL). Similarly, effective inhibition was achieved at low concentrations in *P. damselae* species, but higher concentrations are required for bactericidal effect. Inhibition and bactericidal effect were observed at higher concentrations in *Salmonella Paratyphi* A and *P. mirabilis* species. Especially in *Proteus mirabilis*, the MBC value exceeded 100 mg/mL, indicating reduced bactericidal activity despite the clear inhibitory effect observed with the pure essential oil.

There are various studies in the literature on the antimicrobial activity of juniper EO. Yazgan (2013) conducted a study on the antimicrobial activity of sage EO and its nanoemulsion form on bacteria causing fish spoilage (*P. mirabilis, P. damselae, V. vulnificus*, *Enterococcus faecalis, Pseudomonas luteola* and *Serratia liquefaciens*). MIC and MBC values were analyzed. In the study, *P. luteola* and *S. liquefaciens* species were found to be more sensitive to sage EO compared to other bacteria. Sage EO and its nanoemulsion form showed a high antimicrobial effect on *P. luteola* with a MIC value of 6.25 mg/mL. Similarly, these components showed a strong inhibitory effect on *Proteus mirabilis* with a MIC value of 12.50 mg/mL. However, when the MBC values were analyzed (> 25 mg/mL), it was found that sage EO and nanoemulsion form did not produce a significant bactericidal effect on *Proteus mirabilis*. These results suggest that sage EO has high inhibitory effects, but its bactericidal effects may be more limited. Özoğul et al. (2020) found that grapefruit peel EO showed bacteriostatic properties against most of the bacteria tested in their analysis by disk diffusion, MIC and MBC methods. However, they reported that only the growth of *Salmonella paratyphi* A, *V. vulnificus* and *S. liquefaciens* was inhibited at EO concentrations up to 25 mg/mL. In addition, Rezaei et al. (2016) reported that coriander EO exhibited antimicrobial activity in the range of 2.5–320 μg/mL. Lee et al. (2011) determined the MIC values of *C. indicum* EO in the range of 1.25–10 μg/mL. They also observed that this oil effectively inhibited the growth of bacteria such as *Bacillus cereus, S. aureus, A. hydrophilia, S. choleraesuis, S. enterica, S. sonnei, V. parahaemolyticus* and *V. vulnificus*. They reported the order of MBC as *A. hydrophilia < B. cereus, B. subtilis, V. parahaemolyticus < S. aureus, S. sonnei, V. vulnificus.*

These literature data support that juniper EO shows antimicrobial activity against various bacteria. However, antimicrobial activity may vary depending on the type of bacteria, the type of juniper used and the concentration of application. Studies show that juniper oil is effective against both Gram-positive and Gram-negative bacteria and that these properties are due to terpenic compounds. However, Hammer et al. (1999) reported that essential oils are more effective against Gram-positive bacteria, while Gram-negative bacteria are more resistant due to the cell wall structure. In this study, a similar result was obtained and *S. aureus* was the most sensitive species, while gram-negative *P. mirabilis* was the most resistant species. This may be explained by the fact that juniper EO can target the cell wall of gram-positive bacteria more easily with its chemical components. Juniper EO, which is reported to be particularly effective against fish spoilage bacteria such as *Pseudomonas* spp., *Shewanella* spp. and *Aeromonas* spp. has the potential to extend the shelf life of fresh fish products. Juniper EO nanoemulsions can act on these bacteria by both breaking down the cell wall and disrupting protein and lipid structures. Studies have shown that juniper EO in nanoemulsion form shows high antimicrobial activity even at lower concentrations compared to its free form. This is attributed to the small size and homogeneous distribution of nanoemulsions, as it increases the capacity of the oil to penetrate bacterial cells (Weiss et al., 2009).

In the literature, there are studies examining the effect of essential oils such as thyme, lemon, grapefruit and laurel on pathogenic bacteria. Bhargava et al. (2015) reported the effect of thyme oil nanoemulsions on *Listeria monocytogenes, Salmonella Typhimurium* and *Escherichia coli* O157: H7 and observed a reduction of 3.57, 3.26 and 3.35 log CFU/g at 0.1% concentration, respectively. Between Bhargava et al. (2015) and this study, it is noteworthy that inhibition zone diameters were higher in gram-positive bacteria. However, in this study, it was observed that juniper oil provided similar activity even at lower concentrations. Özoğul et al. (2020) reported MIC values as 1.25 mg/mL and 0.75 mg/mL in studies conducted with nanoemulsions containing thyme oil against *Salmonella Paratyphi* A and *V. vulnificus* bacteria, respectively. In this study, juniper EO showed a high activity with a lower MIC value of 0.5 mg/mL for *V. vulnificus*. These differences are thought to be due to the chemical components of the essential oils used. The components of juniper oil with high antimicrobial properties such as *α*-pinene, sabinene and terpinen-4-ol may explain these differences in efficacy.

The results indicate that juniper essential oil (EO) exhibits strong antimicrobial activity against both Gram-positive and Gram-negative bacteria, particularly when applied in nanoemulsion form. This finding is largely consistent with previous reports on the antibacterial efficacy of essential oils used as natural preservatives. The low MIC and MBC values, together with the wide inhibition zones, clearly demonstrate the potent antimicrobial effect of juniper oil and its nanoemulsion formulations.

The antibacterial activity of juniper nanoemulsions increased consistently in a concentration-dependent manner across all tested species. Although the nanoemulsion formulations exhibited a clear dose-dependent inhibitory effect, pure juniper essential oil showed lower MIC and MBC values under the tested *in vitro* conditions. Collectively, these results emphasize the strong potential of JNEO as an effective natural antimicrobial agent, particularly against Gram-negative marine bacteria associated with fish spoilage.

## 4 Conclusion

This study revealed that nanoemulsion formulations prepared with *Juniperus communis* essential oil at concentrations ranging from 2–6% showed potent and dose-dependent antimicrobial activity against target strains such as *Staphylococcus aureus* and *Vibrio vulnificus*. The nanoemulsion system primarily contributed to improved dispersibility and formulation stability of juniper essential oil, while pure EO exhibited lower MIC and MBC values in vitro. However, the high resistance level of *Proteus mirabilis* showed that the cell wall structure and defence mechanisms of EO should be taken into account when designing a formulation. Considering the EO content of the nanoemulsions, the inclusion of EOs enhanced the bacteriostatic performance of the nanoemulsion system. The findings suggest that *J. communis* essential oil nanoemulsions offer a potential natural alternative in the class of food preservatives. However, for practical applications, evaluation of synergistic interactions of juniper EO with other natural antimicrobials and testing of shelf life, sensory properties and microbial control performance in real food systems should be required. These steps will make it possible to move juniper EO nanoemulsions into industrial applications as both a safe and effective food preservative.

## Data Availability

The raw data supporting the conclusions of this article will be made available by the authors, without undue reservation.
